# The Problem of Anaemia in Patients with Colorectal Cancer

**DOI:** 10.1155/2014/547914

**Published:** 2014-02-12

**Authors:** M. Khanbhai, M. Shah, G. Cantanhede, S. Ilyas, T. Richards

**Affiliations:** Division of Surgery and Interventional Science, University College London, 4th Floor, 74 Huntley Street, London WC1E 6AU, UK

## Abstract

*Background*. Surgical patients are often anaemic preoperatively subsequently requiring blood transfusion. The aim of this study was to assess the problem of anaemia and transfusion rates in patients undergoing surgery for colorectal cancer. *Methods*. Haemoglobin levels and transfusion requirements were assessed retrospectively in 199 sequential patients operated on for colorectal cancer. This was followed by prospective analysis of 147 patients to correlate preoperative anaemia, stage of bowel cancer, and operation performed with rates of blood transfusion and length of hospital stay. *Results*. Preoperatively 44% patients were anaemic retrospectively and 60% prospectively. Anaemia increased the risk of transfusion in both studies (69% anaemic versus 31% nonanaemic, *P* = 0.002 in retrospective series, and 83.7% versus 16.3%, *P* < 0.0001 in prospective series). Anaemia was proportionally higher in patients with Dukes B (65.2%) and Dukes C (66.6%) than in patients with Dukes A (28.5%). Length of stay was prolonged in transfused patients excluding those requiring major blood transfusion (median 13 versus 7 days, *P* < 0.0001). Transfusion was also associated with higher mortality (*P* = 0.05). *Conclusion*. Anaemia is common in patients with colorectal cancer. Anaemic patients were at high risk of receiving blood transfusion, which in turn increased length of stay and mortality.

## 1. Introduction

Anaemia is common in cancer patients with a reported prevalence of 40% in solid tumours [1, 2]. Overall anaemia in patients presenting with cancer is associated with reduced survival [3, 4] and reduced quality of life [2, 5]. Many lower gastrointestinal tumours present as iron deficiency anaemia [6]. Although anaemia is associated with chronic blood loss from the gastrointestinal tract, malignancy induced inflammation and underlying comorbidities are causal factors for anaemia of chronic disease [7]. Treatment whether surgery, chemotherapy, or radiotherapy may further worsen anaemia [7].

In those patients with colorectal cancer undergoing surgical resection, preoperative anaemia was associated with increased risk of perioperative infection, mortality, and a longer inpatient stay [8–13]. Anaemia is also an independent risk factor for allogenic blood transfusion (ABT) with a reported prevalence of in these cohorts of patients of 10%–38% [14]. Transfusion may further worsen outcome following operation. Effective management of anaemia may result in improved functional quality of life and better response to treatment [6, 15–17].

The aim of this study was twofold: firstly, to assess the relationship between preoperative anaemia, tumour stage, and operation performed with transfusion rates and length of hospital stay and, secondly, to assess whether anaemia can be effectively detected preoperatively.

## 2. Methods

**Figure 1:**
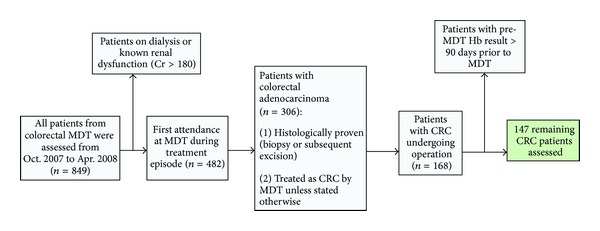
Inclusion and exclusion criteria for patients assessed prospectively from colorectal MDT. CRC, colorectal cancer; Cr, Creatinine; Hb, haemoglobin; MDT, multidisciplinary team.

The study was carried out at a regional colorectal teaching centre in which seven colorectal surgeons serve a population of 2.5 million. In the retrospective series, data on 199 patients with colorectal cancer from 2004 to 2005 was analysed. In the prospective series, data on 147 patients assessed at colorectal cancer multidisciplinary team meeting (MDT) from 2007 to 2008 was analysed (Figure 1). Perioperative transfusion status was obtained from the blood transfusion database. Length of inpatient stay, details of resectional surgery, Dukes stage, and perioperative mortality rates were recorded. In both studies haemoglobin at MDT and admission was assessed. Anaemia was defined as a laboratory haemoglobin concentration of less than 11.5 g/dL for females and 13.0 g/dL for males, values that were the lower limit of normal for each sex. Those patients requiring a major blood transfusion, defined as four or more units of blood transfused, were analysed separately. Major blood transfusion was used as a marker for those patients with significant intraoperative blood loss. The surgeon or anaesthetist assessed the need for perioperative transfusion accordingly.

Data were entered into a Microsoft Excel spreadsheet (Microsoft Office Excel 2007, Microsoft Corporation). Results were reported as mean (SD) or median (range). Fisher's exact test, Wilcoxon rank sum test, *t* test and Mann-Whitney test were used as appropriate. Correlation between anaemia and ABT was studied. Correlation of anaemia and ABT with tumor stage, length of hospital stay, procedure, and mortality was also assessed. Statistical analysis was carried out in SPSS Version 17 (SPSS Inc., Chicago, IL, US).

## 3. Results

In the retrospective series of 199 patients, 52% were male. Average ([±SD] years) age was 72.0 (±11.5). Mean (±SD) preoperative haemoglobin (g/dL) was 12.2 (±2.1). Preoperative anaemia was observed in 88 (44%) patients with mean haemoglobin level 1.9 (±1.6) below the lower limit of normal for sex of that patient. In total 41 patients (20%) were transfused an average of 2.7 units (total 112 units). Of these, 47 units were given preoperatively, 11 intraoperatively, and 54 in the postoperative period. Nine patients received major blood transfusion, where a mean (±SD) of 5 (±0.6) units was given. Anaemia was associated with increased likelihood of transfusion (32% anaemic versus 11% nonanaemic, *P* > 0.0001). With exclusion of major blood transfusion, patients with preoperative anaemia received more blood compared to patients without anaemia (52 versus 15 units, *P* < 0.0001).

**Figure 2:**
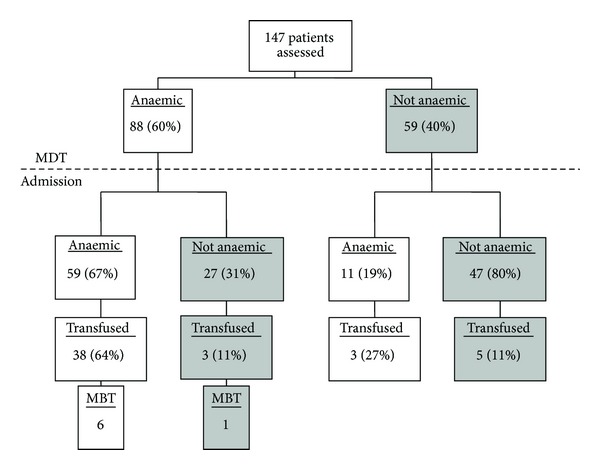
This flowchart describes the incidence of anaemia in colorectal cancer patients undergoing surgery (prospective series). MDT, multidisciplinary team; MBT, major blood transfusion.

In the prospective series, 147 patients were analysed (Figure 2) and 46% were male. Average age ([±SD] years) was 70 (±12). Mean (±SD) admission haemoglobin (g/dL) was 12.1 (±2.4). Anaemia was observed in 70 (47.6%) patients on admission with mean haemoglobin of 2.0 (±1.6) below normal. During admission a total of 109 units of blood were transfused in 42 (29%) patients. Furthermore, a total of 81 units of blood during admission were transfused in 7 (5%) patients who received major blood transfusion. Overall mean length of stay ([±SD] days) was 12 ([±13], 95% CI [10–15]) with a mean interval of 27 ([±50], 95% CI [18–35]) days between assessment at MDT and surgical intervention.

At MDT 88 (60%) patients were anaemic with mean haemoglobin of 1.8 ([±SD] 2.0, 95% CI [1.2–2.4]) below the lower limit of normal for the sex of the patient. Most patients who received blood transfusion were anaemic at MDT (83.7% anaemic versus 16.3% not anaemic, *χ* ^2^ yates = 15.887, d.f = 1, *P* < 0.0001). Those anaemic at MDT (after exclusion of patients receiving major blood transfusion) were transfused more blood (1 unit anaemic versus 0.5 units not anaemic, *P* = 0.0330).

Right-sided colonic resection was carried out in 67 (46%) patients and left-sided resection in 76 (52%). Four patients underwent either total or subtotal colectomy. On subsequent histopathology, 21 (14%) were Dukes A, 46 (31%) Dukes B, and 54 (37%) Dukes C. Patients with Dukes A cancer were least likely to be anaemic; *χ* ^2^ (1) = 10.7, *P* = 0.001 (Table 1). Patients undergoing right-sided procedures were more likely to receive transfusion (38.8%) than patients who underwent left-sided procedures (27.6%) (Table 2). Increased patient age (>65 years) was associated with an increased likelihood of transfusion (*χ* ^2^ yates = 5.033, d.f = 1, *P* = 0.0249).

Overall, anaemia on admission was not related to a longer stay in hospital (median 9 days versus 8 days, *P* = 0.63). However, length of stay was prolonged in transfused patients, excluding those requiring major blood transfusion (median 13 versus 7 days, *P* < 0.0001).

A total of 17 (12%) patients died, a mean (±SD) interval of 68 ([±68], 95% CI [33–103]) days after operation. Twelve (71%) of these patients were anaemic on admission. ABT was undertaken in 11 (65%) of these patients (none received major blood transfusion). ABT was associated with a higher mortality (excluding those receiving major blood transfusion) (*χ* ^2^ yates = 9.297, d.f = 1, *P* = 0.0023). Dukes' stage and type of procedure had no impact on survival (Tables 1 and 2).

## 4. Discussion

We have shown that anaemia is common in patients undergoing resection surgery for colorectal cancer. Our data on incidence of anaemia and association with blood transfusion in patients undergoing surgery are in line with large database analysis in cardiac and noncardiac surgery [8–13]. In our study, anaemia was related to age and tumour stage. Preoperative anaemia and left-sided colonic resection were associated with increased need for blood transfusion. Blood transfusion was independently associated with increased length of stay and increased mortality.

The loco regional response to chemotherapy and radiotherapy may also be impaired in patients with low haemoglobin [6, 15–17]. Anaemia may modify tumour behaviour by promoting more aggressive phenotypes [18]. These factors contribute to high morbidity and mortality observed in cancer patients with perioperative anaemia. High rates of ABT in anaemic patients may be partly responsible for poor outcomes. This hypothesis is supported by reduced survival in patients undergoing ABT in our analyses.

Anaemia was identified well ahead of surgery in our study (mean interval between assessment and admission = 33 days) providing time for preoptimisation. This may improve both survival and quality of life. A haemoglobin improvement of at least 2 g/dL is required to alleviate fatigue and associated symptoms [5]. ABT has been the conventional treatment for perioperative anaemia and is associated with various adverse events. These include transfusion-related lung injury (TRALI), immunomodulation, and haemolytic, allergic and febrile reactions [19–22]. Newly emerging infectious agents also pose a constant threat [23]. There is a possible correlation with tumour growth, recurrence, or disease progression in cancer patients [24]. ABT is associated with advanced age, long duration of operation, massive blood loss, and anemia. ABT is an independent prognostic factor for long-term survival [25], a finding confirmed by our study.

Adoption of patient blood management (PBM) plan can help in early identification of anaemia and patients at high risk of transfusion. This may reduce and eliminate the need for ABT [26]. Management strategies include preoptimisation, restricted phlebotomy, implementation of restrictive transfusion triggers, and refined operative techniques to achieve meticulous haemostasis [26]. Acute normovolemic haemodilution can help increase tolerance for intraoperative anaemia. There is however little evidence to support its efficacy in avoiding ABT [27]. The safety of this approach needs to be addressed adequately.

Anaemia is common in patients with colorectal cancer. This leads to a high risk of ABT, contributing to delayed discharge and increased mortality. Early identification of anaemia and preoptimisation may help in reducing the requirements for blood transfusion.

## Figures and Tables

**Table 1:** Relationship between Dukes stage and anaemia, transfusion, and mortality.

Dukes stage		Anaemia		Transfusion		Mortality	
	*n*	*n*_1_	%	*n*_2_	%	*n*_3_	%
A	21	6	28.5%	5	23.8%	2	9.5%
B	46	30	65.2%	15	32.6%	4	8.6%
C	54	36	66.6%	18	33.3%	7	12.9%

**Table 2:** Relationship between type of procedure and anaemia as well as transfusion and mortality.

Procedure		Anaemia		Transfusion		Mortality	
	*n*	*n*_1_	%	*n*_2_	%	*n*_3_	%
Right	67	44	65.6%	26	38.8%	8	11.9%
Left	76	41	53.9%	21	27.6%	9	11.8%
Other	4	3	75%	2	50%	0	0%
